# Functional polymorphisms in FOXC2 gene and Epithelial ovarian Cancer susceptibility in Chinese population

**DOI:** 10.1186/s13048-020-00634-7

**Published:** 2020-03-28

**Authors:** Zhijiao Zhou, Xiang Ou, Qiong Zou, Ling Chu, Xiyun Quan, Yong Chen, Yang Liu

**Affiliations:** 1 Department of Pathology, Third Xiangya Hospital,Central South University, Changsha, 410013 Hunan China; 2 Department of Endocrinology, The First Hospital of Changsha, Changsha, Hunan China; 3 Department of Pathology, Zhuzhou Central Hospital, Zhuzhou, Hunan China; 4 Department of Clinical Laboratory, The First Hospital of Changsha, Changsha, Hunan China

**Keywords:** Forkhead box protein C2, Epithelial ovarian cancer, Polymorphism

## Abstract

**Background:**

Epithelial ovarian cancer (EOC) is highly lethal gynecological cancer. Forkhead Box Protein C2 (FOXC2) promotes occurrence and development of various malignant tumors. The present study is aimed at exploring the correlation between the polymorphism of FOXC2 and epithelial ovarian cancer susceptibility in Chinese Han population.

**Methods:**

A case-control design was used to verify the association between FOXC2 polymorphisms and epithelial ovarian cancer. The genotyping was performed using Taqman® SNP Genotyping kit by qRT-PCR. The genetic variants including rs3751794 C > T, rs1035550 A > G, rs4843163 C > G and rs4843396 C > T in FOXC2 gene were analyzed. The strength of the associations was detected using odds ratios and 95% confidence intervals. Stratification analyses showed the association between the FOXC2 gene polymorphisms rs3751794 C > T, rs4843163 C > G and rs4843396 C > T with epithelial ovarian cancer susceptibility in terms of age, metastasis status, clinical stage, pathological grade, pregnant times, pausimenia, and the expression of ER, PR, wild p53 and mutant p53.

**Results:**

Rs3751794 C > T (*P* = 0.0016), rs4843163 C > G (*P* < 0.0001) and rs4843396 C > T (*P* < 0.0001) were significantly associated with increased epithelial ovarian cancer risk. In stratification analyses,rs3751794 C > T, was identified to be dominant in no metastasis patients, clinical stage 4 group, middle grade pathological stage, pregnant time over 3 patients, post-menopause women, strong wild type p53 expression; rs4843163 C > G was dominant in high grade clinical stage, high grade pathological stage, post-menopause women, strong ER expression group and no mutant p53 expression group; rs4843396 C > T was dominant in high grade clinical stage, high grade pathological stage, strong ER expression group. The rs1035550 A > G was not related to epithelial ovarian cancer susceptibility.

**Conclusions:**

The results of the current study verified that FOXC2 gene polymorphisms were associated with increased epithelial ovarian cancer risk and suggested that FOXC2 gene polymorphisms might be a potential biomarker for epithelial ovarian cancer susceptibility.

## Introduction

Ovarian cancer is the sixth malignant tumor in women. Since it almost doesn’t show specific symptoms at early stage, it is usually diagnosed very late, even when tumor cells spread or metastasis [1]. In 2018, it’s estimated there were more than 295 thousands new cases and 184 thousands deaths of ovarian cancer over the world [2]. Epithelial ovarian cancer (EOC) is the dominant type of ovarian cancer. The standard treatment for EOC is cytoreductive surgery combined with chemotherapy. However, most EOC patients relapse and the 5-year survival rate is no more than 35% [3]. The unsatisfying outcome of EOC treatment is attributed to late diagnosis and chemotherapy resistance [4]. Hence, there is urgent need for revealing risk factors which can be used for early diagnosis.

Several evidence from genome-wide association study (GWAS) showed that genetic variations were identified to associate with ovarian cancer, most of which were single nucleotide polymorphism. Some genetic variants were discovered to be shared between East Asian and European populations, but still others were specific in each population [5]. In Chinese population, genes such as WNT4 [6], PSEN1, MAML2 [7], ESR2 [8], et al. were identified to be associated with EOC.

Forkhead Box Protein C2 (FOXC2), which is an important member of transcription factor FOX family, plays essential role in several gene regulatory pathways. It was also showed to play role in carcinogenesis. FOXC2 was identified to induce epithelial-mesenchymal transition (EMT) in prostate cancer [9],lung cancer [10]. Overexpression of FOXC2 was related to poor prognosis of hepatocellular carcinoma [11].FOXC2 activated YAP signal pathway and enhanced the glycolysis in nasopharyngeal carcinoma cells [12]. Still FOXC2 was certificated to promote EMT, migration and invasion in cisplatin-resistant ovarian cancer cells [13]. Mutations of DNA binding domain in FOXC2 were identified to induce Lymphoedema distichiasis syndrome [14]. It’s reported that FOXC2 polymorphism was associated with type 2 diabetes mellitus [15]. FOXC2 c.-512C > T was found to related with increased susceptibility to chronic venous diseases [16]. However, there was no evidence showed that FOXC2 polymorphisms were associated with cancers.

This study focused on the relevance between the polymorphism of FOXC2 and epithelial ovarian cancer susceptibility.

## Materials and methods

### Study populations

A total of 150 epithelial ovarian cancer cases and 298 healthy controls from The Third Affiliated Hospital, Central South University were included in this study. The major clinical and biological characteristics of the patient, including age, metastasis status, clinical stage, pathological grade, pregnant times, pausimenia, and the expression of ER, PR, wild p53 and mutant p53 by immunohistochemistry (IHC) were collected. There was no significant difference in age between the case group and the control group. Ethical approved was obtained from the Institutional Review Board of the hospital.

### SNP genotyping and quality control

The included potentially functional candidate SNPs were selected as follows: located in the exon, 5’flanking region, 5’untranslated region, and 3′ untranslated region of FOXC2 gene. NCBIdb SNP database (http://www.ncbi.nlm.nih.gov/projects/SNP) and SNPinfo (http://snpinfo.niehs.nih.gov/snpfunc.htm) online software were used to selected SNPs [17]. We chose four potentially functional SNPs in FOXC2 gene (rs3751749 C > T, rs1035550 A > G, rs4843163 C > G and rs4843396 C > T) for analysis. All of the four SNPs mapped in 5’untranslated region, and predicted to act as transcription factor binding sites. Genomic DNA was derived from paraffin embedding tissues or EDTA peripheral blood by using DNA extracting Kit (TianGen Biotech Co. Ltd., Beijing, China). The genotyping of all the subjects was carried out using TaqMan real-time PCR (Applied Biosystems), according to the manufacturer’s protocols. The quality control was performed as the protocol from published paper [18].

### Statistical analyses

Departures from Hardy-Weinberg equilibrium (HWE) were evaluated for each SNP in controls by goodness-of-fit χ^2^ test. Two-sidedχ^2^ test and *t* test were performed, as appropriate to compare the demographic variables and allele frequencies between the cases and the control group. The odds ratio (OR), and the corresponding 95% confidence interval (CI) for each SNP were analyzed. Logistic regression analysis was used to assess the correlation between SNPs and epithelial ovarian cancer susceptibility. Statistical adjustment for age was performed. The version 9.4 SAS software (SAS Institute, Cary, NC) was used to perform analysis. The significant threshold was *P* < 0.05.

## Results

### Population characteristics

There was no significant difference in terms of age (*P* = 0.252) between the case and the control groups. Of them, 18, 27, 77, and 24 patients were classified as clinical stages I, II, III, and IV; 20, 32 and 92 patients were classified as pathological grades low, middle and high, respectively. Among these cases, 93 patients were postmenopausal, 76 patients had metastasis. According to IHC detection, the expression of ER in 4 patients was negative/mild positive, in 40 patients was strong positive; the expression of PR in 16 patients was negative/mild positive, in 32 patients was strong positive; the expression of wild type p53 in 37 patients was negative/mild positive, in 29 patients was strong positive; the expression of mutant p53 in 24 patients was negative/mild positive, in 36 patients was strong positive.

### Correlation of FOXC2 gene polymorphisms with epithelial ovarian cancer susceptibility

The genotype frequencies of FOXC2 associated with epithelial ovarian cancer risk were shown in Table 1.Because rs1035550 G > A was not accordance with HWE (< 0.05), we would not analyze the relationship between rs1035550 and epithelial ovarian cancer risk further. A positive association between rs3751749 C allele and epithelial ovarian cancer risk (TC vs. TT: adjusted OR = 3.415, 95% CI = 1.492–7.815, *P* = 0.0036; recessive model: adjusted OR = 3.707, 95% CI = 1.645–8.353, *P* = 0.0016). A positive association between rs4843163 C allele and epithelial ovarian cancer risk (GC vs. GG: adjusted OR = 20.567, 95% CI = 10.522–40.202, *P* < 0.001; CC vs.GG: adjusted OR = 1.691, 95% CI = 1.034–2.765, *P* = 0.0363; dominant model: adjusted OR = 3.703, 95% CI = 2.408–5.693, *P* < 0.0001; recessive model: adjusted OR = 15.831, 95% CI = 8.449–29.664, *P* < 0.0001). A positive association between rs4843396 C allele and epithelial ovarian cancer risk (TC vs. TT: adjusted OR = 20.567, 95% CI = 10.522–40.202, *P* < 0.001; CC vs. TT: adjusted OR = 1.691, 95% CI = 1.034–2.765, *P* = 0.0363; dominant model: adjusted OR = 3.628, 95% CI = 2.358–5.582, *P* < 0.0001; recessive model: adjusted OR = 23.546, 95% CI = 14.555–44.837, *P* < 0.0001).

**Table 1:**
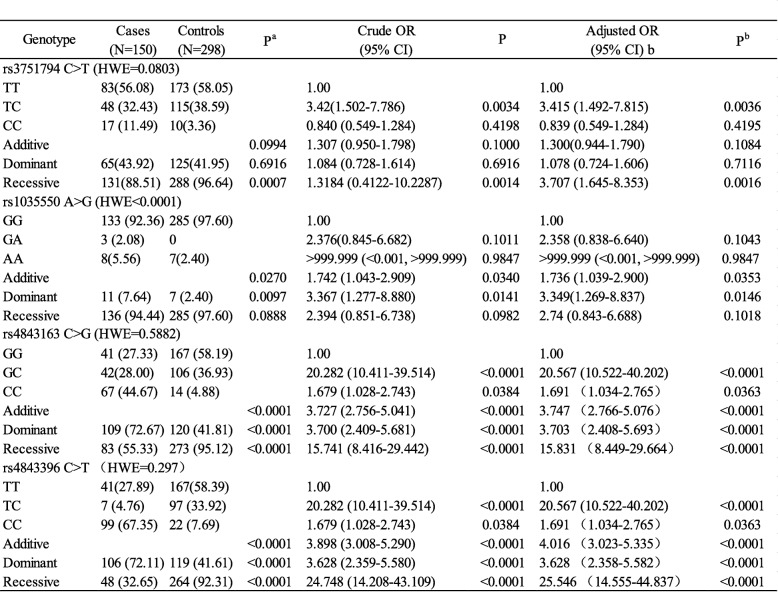
Logistic Regression Analysis of Associations Between FOXC2 Polymorphisms and EOC susceptibility

### Stratification analysis

The results were showed in Table 2 from stratification analyses of association between FOXC2 genotypes and epithelial ovarian cancer susceptibility, stratified by age, metastasis status, clinical stage, pathological grade, pregnant times, pausimenia, the expression of ER, PR, wild p53 and mutant p53. For age, FOXC2 rs3751794 CC genotype was significantly associated with increased epithelial ovarian cancer risk in both< 53 years group (adjusted OR = 5.289;95% CI = 1.331–21.013, *P* = 0.0180) and ≥ 53 years group (adjusted OR = 3.010;95% CI = 1.089–8.322, *P* = 0.0337); rs4843163GC/CC genotype was significantly associated with increased epithelial ovarian cancer risk in both< 53 years group (adjusted OR = 2.761;95% CI = 1.600–4.764, *P* = 0.0003) and ≥ 53 years group (adjusted OR = 5.844;95% CI = 2.585–12.115, *P* < 0.0001); rs4843396TC/CC genotype was significantly associated with increased epithelial ovarian cancer risk in both< 53 years group (adjusted OR = 3.239;95% CI = 1.854–5.659, *P* < 0.0001) and ≥ 53 years group (adjusted OR = 4.272;95% CI = 2.160–8.447, *P* < 0.0001).Z

**Table 2:**
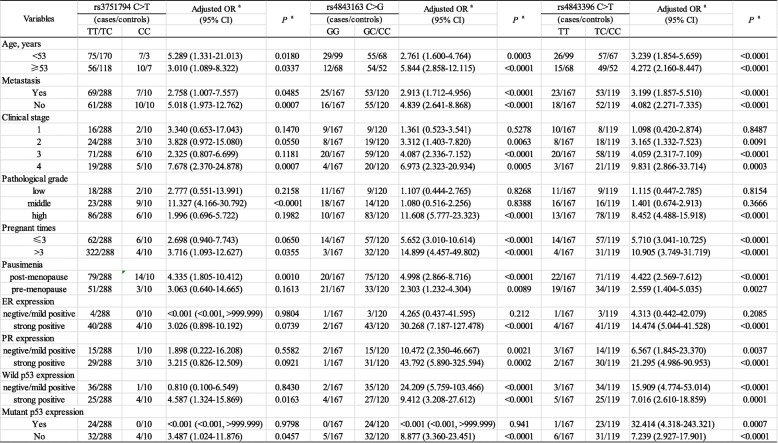
Stratification analysis of FOXC2 polymorphisms with EOC susceptibility

For metastasis status, FOXC2 rs3751794 CC genotype was significantly associated with increased epithelial ovarian cancer risk in no metastasis group (adjusted OR = 5.018;95% CI = 1.973–12.762, *P* = 0.0007); rs4843163GC/CC genotype was significantly associated with increased epithelial ovarian cancer risk in subgroups of metastasis (yes group: adjusted OR = 2.913;95% CI = 1.712–4.956 *P* < 0.0001; no group: adjusted OR = 4.839;95% CI = 2.641–8.868, *P* < 0.0001); rs4843396TC/CC genotype was significantly associated with increased epithelial ovarian cancer risk in subgroups of metastasis (yes group: adjusted OR = 3.199;95% CI = 1.857–5.510 *P* < 0.0001; no group: adjusted OR = 4.082;95% CI = 2.271–7.335, *P* < 0.0001).

For clinical stage, FOXC2 rs3751794 CC genotype was significantly associated with increased epithelial ovarian cancer risk in stage 4 patients (adjusted OR = 7.678;95% CI = 2.730–24.878, *P* = 0.0007);rs4843163GC/CC genotype was significantly associated with increased epithelial ovarian cancer risk in three subgroups of clinical stage (stage 2: adjusted OR = 3.312;95% CI = 1.403–7.820, *P* = 0.0063; stage 3: adjusted OR = 4.087;95% CI = 2.336–7.152, *P* < 0.0001; stage 4: adjusted OR = 6.973;95% CI = 2.323–20.934, *P* = 0.0005); rs4843396TC/CC genotype was significantly associated with increased epithelial ovarian cancer risk in three subgroups of clinical stage (stage 2: adjusted OR = 3.165;95% CI = 1.332–7.523, *P* = 0.0091; stage 3: adjusted OR = 4.059;95% CI = 2.317–7.109, *P* < 0.0001; stage 4: adjusted OR = 9.831;95% CI = 2.866–33.714, *P* = 0.0003).

For pathological grade, rs3751794 CC genotype was significantly associated with increased epithelial ovarian cancer risk in middle grade group (adjusted OR = 11.327; 95% CI = 4.166–30.792, *P* < 0.0001); rs4843163GC/CC genotype (adjusted OR = 11.608;95% CI = 5.777–23.323, *P* < 0.0001) and rs4843396TC/CC genotype (adjusted OR = 8.452;95% CI = 4.488–15.918, *P* < 0.0001) was significantly associated with increased epithelial ovarian cancer risk in high grade group.

For pregnant times, rs3751794 CC genotype was significantly associated with increased epithelial ovarian cancer risk in > 3 times group (adjusted OR = 3.716;95% CI = 1.093–12.627, *P* = 0.0355); rs4843163GC/CC genotype was significantly associated with increased epithelial ovarian cancer risk in subgroups of metastasis (≤3 times group: adjusted OR = 5.652;95% CI = 3.010–10.614 *P* < 0.0001; > 3 times group: adjusted OR = 14.899;95% CI = 4.457–49.802, *P* < 0.0001); rs4843396TC/CC genotype was significantly associated with increased epithelial ovarian cancer risk in subgroups of metastasis (≤3 times group: adjusted OR = 5.710;95% CI = 3.041–10.725 *P* < 0.0001; > 3 times group: adjusted OR = 10.905;95% CI = 3.749–31.719, *P* < 0.0001).

For pausimenia, rs3751794 CC genotype was significantly associated with increased epithelial ovarian cancer risk in postmenopausal patients (adjusted OR = 4.338;95% CI = 1.805–10.412, *P* = 0.0010); rs4843163GC/CC genotype was significantly associated with increased epithelial ovarian cancer risk in subgroups of pausimenia (yes group: adjusted OR = 4.998;95% CI = 2.866–8.716 *P* < 0.0001; no group: adjusted OR = 2.303;95% CI = 1.232–4.304, *P* = 0.0089); rs4843396TC/CC genotype was significantly associated with increased epithelial ovarian cancer risk in subgroups of metastasis (yes group: adjusted OR = 4.422;95% CI = 2.569–7.612 *P* < 0.0001; no group: adjusted OR = 2.559;95% CI = 1.404–5.035, *P* = 0.0027).

Furthermore, we identified that rs4843163GC/CC genotype (adjusted OR = 30.268; 95% CI = 7.187–127.478, *P* < 0.0001) and rs4843396TC/CC genotype (adjusted OR = 14.474;95% CI = 5.044–41.528, *P* < 0.0001) was remarkably associated with strong positive ER expression. These two FOXC2 polymorphisms were significantly associated with subgroups of PR expression (rs4843163GC/CC vs. GG: negative/mild expression: adjusted OR = 10.472;95% CI = 2.350–46.667, *P* = 0.0021; strong positive expression: adjusted OR = 43.792;95% CI = 5.890–325.594, *P* = 0.0002; rs4843396TC/CC vs. TT: negative/mild expression: adjusted OR = 6.567;95% CI = 1.845–23.370, *P* = 0.0037; strong positive expression: adjusted OR = 21.295;95% CI = 4.986–90.953, *P* < 0.0001). For wild type p53 expression, rs3751794 CC genotype was significantly associated with increased epithelial ovarian cancer risk in strong positive group (adjusted OR = 4.587;95% CI = 1.324–15.869, *P* = 0.0163); rs4843163GC/CC genotype was significantly associated with increased epithelial ovarian cancer risk in subgroups of wild type p53 expression (negative/mild expression: adjusted OR = 24.209;95% CI = 5.759–103.466, *P* < 0.0001; strong positive expression: adjusted OR = 9.412;95% CI = 3.208–27.612, *P* < 0.0001); rs4843396TC/CC genotype was significantly associated with increased epithelial ovarian cancer risk in subgroups of wild type p53 expression (negative/mild expression: adjusted OR = 15.909;95% CI = 4.774–53.014, *P* < 0.0001; strong positive expression: adjusted OR = 9.412;95% CI = 3.208–27.612, *P* = 0.0001). For mutant p53 expression, rs3751794 CC genotype (adjusted OR = 3.487;95% CI = 1.024–11.876, *P* = 0.0457 and rs4843163GC/CC genotype (adjusted OR = 8.877;95% CI = 3.360–23.451, *P* < 0.0001) was significantly associated with increased epithelial ovarian cancer risk in strong positive group; rs4843396TC/CC genotype was significantly associated with increased epithelial ovarian cancer risk in subgroups of mutant p53 expression (negative/mild expression: adjusted OR = 32.414;95% CI = 4.318–243.321, *P* = 0.0007; strong positive expression: adjusted OR = 7.239;95% CI = 2.927–17.901, *P* = 0.0001).

## Discussion

In the current case-control study with 150 epithelial ovarian cancer cases and 298 healthy controls from Chinese populations, we explored the potential association between FOXC2 gene polymorphisms and epithelial ovarian cancer susceptibility. We certificated that 3 of the 4 included polymorphisms, namely rs3751749 C > T, rs4843163 C > G and rs4843396 C > T, were associated with an increased risk of epithelial ovarian cancer. So far, the current study is the first to evaluate the association between FOXC2 polymorphisms and epithelial ovarian cancer susceptibility.

Mutations of FOXC2 in coding region could impair its transcriptional activation and DNA-binding activity in hereditary distichiasis [19, 20]. A nonsense mutation in exon was found in spinal extradural arachnoid cyst [21]. Deletion of FOXC2 could induce abnormal lymphagiogenesis and activate ERK [22]. Yamada Y, et al. revealed that FOXC2 polymorphism was associated with reduced BMD susceptibility in Japanese population [23]. In addition to these diseases, there was no evidence shown mutation of FOXC2 in other diseases.

In this study, we analyzed the correlation between FOXC2 polymorphisms and the patient’s metastasis status, clinical stage, pathological grade, pregnant times, pausimenia, and the expression of several EOC related proteins. And we discovered that rs3751749 C > T, rs4843163 C > G and rs4843396 C > T were associated with increased EOC risk. There is still no evidence shown that FOXC2 polymorphism is associated with cancer, even though in ovarian cancer. All of the three SNPs we detected are in upstream of FOXC2 gene. They were predicted to be function with transcription factors. In this study, we still don’t know whether rs3751749, rs4843163 of rs4843396 could affect the expression of FOXC2. In our next study, we need collect much larger sample sizes to certify the results of the current study, and the effects of these three SNPs on the expression of FOXC2 need to be discovered further in EOC cell lines.

With regard to another FOXC2 polymorphism rs1035550 A > G, we couldn’t find the correlation between this SNP and EOC susceptibility. Rs1035550 locates in upstream of FOXC2. There is also no data shown the correlation between rs1035550 and cancers. Still no result was shown the influence of rs1035550 on the expression of FOXC2.

Although to our knowledge this study is the first to reveal the relationship between FOXC2 polymorphisms and EOC susceptibility, several limitations should be mentioned. Firstly, there were only four SNPs were investigated in the current study, more potentially functional SNPs in FOXC2 gene should be focused on. Secondly, the sample size should be enlarged. Thirdly, patients and healthy control should be enrolled from more hospitals to avoid selection bias.

From this study, we observed that FOXC2rs3751749 C > T, rs4843163 C > G and rs4843396 C > T were associated with increased ovarian cancer risk in Chinese population.
